# Intramyocardial injection of human adipose-derived stem cells ameliorates cognitive deficit by regulating oxidative stress–mediated hippocampal damage after myocardial infarction

**DOI:** 10.1007/s00109-021-02135-6

**Published:** 2021-10-11

**Authors:** Tsung-Ming LEE, Cheng-Che LEE, Horng-Jyh HARN, Tzyy-Wen Chiou, Ming-Hsi CHUANG, Chun-Hung CHEN, Chi-Hsuan CHUANG, Po-Cheng LIN, Shinn-Zong LIN

**Affiliations:** 1 Good Heart Clinic, Tainan, Taiwan; 2 Kang-Ming Senior High School, Tainan, Taiwan; 3 Bioinnovation Center, Tzu Chi Foundation, Department of Pathology, Buddhist Tzu Chi General Hospital, Tzu Chi University, Hualien, Taiwan; 4 Department of Life Science and Graduate Institute of Biotechnology, National Dong Hwa University, Hualien, Taiwan; 5 Department of Technology Management, Chung Hua University, Hsinchu City, Taiwan; 6 Gwo Xi Stem Cell Applied Technology, Hsinchu, Taiwan; 7 Genomics Research Center, Academia Sinica, Taipei, Taiwan; 8 Bioinnovation Center, Tzu Chi Foundation, Department of Neurosurgery, Buddhist Tzu Chi General Hospital, Tzu Chi University, No.707, Sec. 3, Chung Yang Rd. 970, Hualien, Taiwan

**Keywords:** Cognitive function, Hippocampus, Human adipose-derived stem cells, Myocardial infarction, Passive avoidance test, Reactive oxygen species

## Abstract

**Abstract:**

Cognitive impairment is a serious side effect of post-myocardial infarction (MI) course. We have recently demonstrated that human adipose-derived stem cells (hADSCs) ameliorated myocardial injury after MI by attenuating reactive oxygen species (ROS) levels. Here, we studied whether the beneficial effects of intramyocardial hADSC transplantation can extend to the brain and how they may attenuate cognitive dysfunction via modulating ROS after MI. After coronary ligation, male Wistar rats were randomized via an intramyocardial route to receive either vehicle, hADSC transplantation (1 × 10^6^ cells), or the combination of hADSCs and 3-Morpholinosydnonimine (SIN-1, a peroxynitrite donor). Whether hADSCs migrated into the hippocampus was assessed by using human-specific primers in qPCR reactions. Passive avoidance test was used to assess cognitive performance. Postinfarction was associated with increased oxidative stress in the myocardium, circulation, and hippocampus. This was coupled with decreased numbers of dendritic spines as well as a significant downregulation of synaptic plasticity consisting of synaptophysin and PSD95. Step-through latency during passive avoidance test was impaired in vehicle-treated rats after MI. Intramyocardial hADSC injection exerted therapeutic benefits in improving cardiac function and cognitive impairment. None of hADSCs was detected in rat’s hippocampus at the 3rd day after intramyocardial injection. The beneficial effects of hADSCs on MI-induced histological and cognitive changes were abolished after adding SIN-1. MI-induced ROS attacked the hippocampus to induce neurodegeneration, resulting in cognitive deficit. The remotely intramyocardial administration of hADSCs has the capacity of improved synaptic neuroplasticity in the hippocampus mediated by ROS, not the cell engraftment, after MI.

**Key messages:**

- Human adipose-derived stem cells (hADSCs) ameliorated injury after myocardial infarction by attenuating reactive oxygen species (ROS) levels.
- Intramyocardial administration of hADSCs remotely exerted therapeutic benefits in improving cognitive impairment after myocardial infarction.
- The improved synaptic neuroplasticity in the hippocampus was mediated by hADSC-inhibiting ROS, not by the stem cell engraftment.

**Supplementary Information:**

The online version contains supplementary material available at 10.1007/s00109-021-02135-6.

## Introduction

Myocardial infarction (MI) has been associated with cognitive impairment [1, 2]. Previous studies have shown that cerebral blood flow decreases at the onset of MI and remains lower than baseline for 30 days [3]. Increased reactive oxygen species (ROS) were interconnected between cardiac and brain [4]. Epidemiological studies have found that the incidence of cognitive dysfunction after MI was approximately 10–15%, 3–5 times greater than those of patients without coronary heart disease [2]. Survivors of MI may experience continued functional decline over the years after the event [2]. The population of MI survivors is substantial and growing. A better understanding of cognitive disability after MI may improve the therapeutic outcomes.

Excessive ROS production is known to promote cardiovascular diseases, and this may have a neurotoxic effect with regard to brain plasticity. We have demonstrated that post-MI was associated with increased ROS release [5, 6]. ROS caused by MI accumulates in different organs, which may persistently destroy the cells and lead to diseases. In the acute phase of MI, local inflammatory responses in the myocardium and cytokine/chemokine secretion occur concomitantly with the systemic release of ROS [7]. The brain may be at a high risk of free radical–induced oxidative stress, because of the lower amount of antioxidant enzymes, the high rate of oxidative metabolic activity using 20% of the total body oxygen, and the high membrane surface area to cytoplasmic volume ratio [8, 9]. MI has been shown to induce a significant increase of ROS in the hippocampus [7]. The hippocampus has been shown to be the area of the brain at highest risk of oxidative stress [10], and consequently to have the highest risk of functional decline. The dentate gyrus and cornu ammonis areas of the hippocampus have been shown to exhibit synaptic plasticity [11]. Moreover, elevated levels of ROS in specific regions of the dentate gyrus have been shown to lead to significant functional consequences. This is particularly significant as the dentate gyrus has a preferential role in learning and memory function. Rat subjected to high ROS showed marked deficits in learning and memory functions [12]. Exogenous administration of superoxide results in transient reduction in postsynaptic response, followed by impaired long-term potentiation, which can be rescued by superoxide dismutase application [13].

Synapses are generally accepted to play key roles in neuronal networks and information transmission, and neuronal synaptic plasticity has been shown to be important for learning and memory ability [14, 15]. Synaptophysin is a membrane protein that is distributed in almost all pre-synaptic terminals, and it has been demonstrated to be involved in the formation of synapses, the release of neurotransmitters, and the synaptic vesicle cycle [16, 17]. The mice with synaptophysin knockout have been shown to be associated with impaired hippocampal integrity which leads to deficits in learning and memory [18].

The regeneration of tissue using stem cells has shown promise with regard to improved treatment options. Intramyocardial administration of human adipose-derived stem cells (hADSCs) has been shown to be promising treatment to improve functional outcomes after MI [6, 19]. We have demonstrated that hADSC transplantation has been shown to be an effective regenerative therapy for functional and anatomical improvement in animal models of MI [6, 19]. It remained unclear whether intramyocardial hADSC transplantation could remotely regulate the brain and alleviate cognitive impairment after MI. Although the local transplantation of neural stem cells has been reported to improve learning and memory function [20], significant challenges in the application of such therapies for neurological conditions include difficulty in delivering stem cells to the brain and subsequent damage to intact brain tissues. Cao et al. [21] have shown that intraperitoneal administration of human umbilical cord–derived mesenchymal stem cells improved cognitive function in a murine model of aging although the human nuclear antigen was not detectable in the mice brain. It implies that remote transplantation of stem cells alleviated neuropathology via soluble factors derived from stem cells could enter into the circulation system and then play important roles. We and others have shown that soluble factors released from ADSCs contain antioxidation mediators that lead to the release of growth factors and scavenge ROS, which in turn protects fibroblasts from oxidative stress and activates cell membrane receptors and increases glutathione and superoxide dismutase activity [22–24]. In this study, we assessed (1) whether intramyocardial administration of hADSCs can alleviate cognitive impairment; (2) whether intramyocardial transplanted hADSCs migrated remotely away from the implantation sites to the brain, albeit outside the heart; and (3) the role of hADSCs in attenuating ROS-mediated hippocampal injury in rats with MI.

## Materials and methods

All animal and cell culture experiments were conducted according to the local guidelines for the care and use of laboratory animals and approved by the hospital ethics committee (CMUIACUC-2018–361). In addition, the experiments were conducted in accordance with the *Guide for the Care and Use of Laboratory Animals* (US National Institutes of Health, Publication No. 85–23, revised 1996).

### Isolation of hADSCs

hADSCs were purchased from the commercially available kits and were generously provided by Gwo Xi Stem Cell Applied Technology (Hsinchu, Taiwan) as previously described [6, 22]. The cell viability of ADSCs was evaluated by analyzing DNA binding fluorophores. Detailed information is provided in the online Supplementary material.

### Characterization of hADSC surface phenotype

Surface immunophenotye of hADSCs was examined. Detailed information is provided in the online Supplementary material.

### Animals

MI was induced in 8-week-old male Wistar rats by ligating the anterior descending artery as described previously [6]. A ventilator (Harvard Apparatus 486) was used to provide the animals with 95% O_2_ and 5% CO_2_. For surgery, hemodynamic measurements, and sacrifice, rats were intraperitoneally anaesthetized with Zoletil (20 mg/kg body weight) and xylazine (9 mg/kg). The level of anesthesia was checked according to reflexes of the hind feet before and during the procedures, respiratory patterns, and responses to manipulations.

One hour after ligation, rats were assigned into 3 groups with the following treatments via an intramyocardial route: vehicle, hADSC transplantation (1 × 10^6^ cells), or hADSC transplantation + SIN-1 (1 mg/kg per day, 3-Morpholinosydnonimine, a peroxynitrite donor). In the cell transplantation procedure, hADSCs were removed from the plates, suspended in PBS (30 μL; 1 × 10^6^ cells), and transplanted into viable myocardium around the infarction at three injection sites using a 30-gauge syringe needle. The dose of SIN-1 diluted in PBS was used as previously described [25]. Sham-operated rats served as controls. The rats were sacrificed 3 or 30 days post-MI to represent the early and late stages of MI, respectively.

### Passive avoidance test

The passive avoidance test based on contextual-fear conditioning is used to evaluate short-term memory and consists of three parts: (1) exploration test, (2) acquisition test, and (3) retention test. Each rat underwent passive avoidance test at the 29th and 30th day after MI.

#### Exploration test

The rats each performed three exploration tests on the same day. Two compartments (one light and one dark) were created in one chamber (Passive Avoidance Set-up, Ugo Basile, Italy). Each compartment had a stainless-steel bar floor, and both compartments were separated by a guillotine door. An animal was then placed in the light compartment and allowed to explore for 3 min.

#### Acquisition test

After the exploration trial, the door was opened so the rat could move into the dark compartment. The door was lowered and a 0.3-mA, 1-s shock was delivered to the feet of the rats twice at 5-s intervals. This was then repeated 2 min later. The rat received the shock each time it placed all four paws in the dark compartment.

#### Retention test

The rats performed the retention test 24 h after the acquisition test. The time that the rat took to enter the dark compartment was defined as step-through latency. During the test, no electric shock was applied. If the rat did not enter the dark compartment within 300 s, a latency of 300 s was recorded and the rat was returned to its cage.

### Hemodynamics and infarct size measurements

At the end of the passive avoidance test (30 days after MI), the rats were anesthetized and hemodynamic parameters were measured via a polyethylene Millar catheter inserted into the LV and connected to a transducer. Only rats with large infarctions (> 30%) were analyzed. Detailed information is provided in the online Supplementary material.

### Assessment of hADSCs in the myocardium

To determine whether hADSCs retained in the heart, the myocardium was analyzed by immunohistochemistry specific for human mitochondria (1:100, Chemicon International) according to the manufacturer’s instructions. The sections were subsequently quantitatively evaluated. In control studies, the primary antibodies were substituted with 5% normal goat serum in PBS.

### In situ detection of superoxide anion in the heart and brain

Intracellular superoxide production in the dentate gyrus and myocardium regions was evaluated using in situ dihydroethidium (DHE). Detailed information is provided in the online Supplementary material.

### Hippocampal histological assessment

At the end of passive avoidance test, whole brains were removed and the hippocampi were dissected, post-fixed in 4% paraformaldehyde in 0.1 M PBS (pH 7.4) overnight, placed in 30% sucrose/PBS at 4 °C, and then standard frozen sections were generated. The tissues were embedded in paraffin and a series of slides were sampled.

Nissl staining, a neuron marker, was used to stain nucleic acid. The cell layer of the dentate gyrus region was captured using a digital microscope camera. Positively Nissl-stained cells were expressed as the number of cells per area selected in the region of interest.

Golgi staining was used to examine the density of dendritic spines in the dentate gyrus according to the manufacturer’s instructions (Hito Golgi–Cox OptimStain PreKit, Hitobiotec, Kingsport, TN, USA). Spine density was calculated as the number of spines per micrometer. Numbers of positive-stained dendritic spines were counted in a blinded manner with Image Pro Plus software (Media Cybernetics, Silver Spring, USA).

To determine the numbers of presynaptic and postsynaptic puncta, we measured synaptophysin and postsynaptic density protein 95 (PSD95) intensities by immunohistochemical analysis. The hippocampal dentate gyrus sections were incubated with rabbit anti-synaptophysin antibody (1:200, Abcam, Cambridge, MA, USA) and anti-PSD95 antibody (1:200, Abcam, Cambridge, MA, USA) at 4 °C. The secondary antibodies were goat anti-mouse IgG-FITC for synaptophysin and goat anti-rabbit IgG-TRITC for PSD95. Negative controls were done by replacing the primary antibody with the antibody diluent.

### Western blot of synaptophysin and PSD95

Tyrosine phosphorylation of synaptophysin has been shown to be necessary for long-term potentiation, a cellular basis of learning and memory [26]. Rat hippocampal was dissected after snap frozen. Western blots were performed and analyzed as previously described [6]. Antibodies used for Western blots were as follows: synaptophysin (Abcam, Cambridge, MA, USA) and rabbit anti-PSD95 antibody (Abcam), and β-actin (Cell Signaling Technology).

### qPCR of human-specific β-2-microglobulin

To trace whether hADSCs migrated into the hippocampus, quantitative PCR (qPCR) was used by specific primers for human sequence for β-2-microglobulin. Detailed information is provided in the online Supplementary material.

### Laboratory measurements

To confirm the systemic effect of MI on circulating ROS levels, we measured plasma superoxide levels. The nitro blue tetrazolium reaction in TRIS-buffer was used to determine the plasma superoxide level [27, 28] 3 days after MI. As the reduction can be inhibited by superoxide dismutase, this reaction is specific for superoxide radicals [29]. All measurements were performed at a wavelength of 530 nm.

Tissue superoxide production from the myocardial border zone and hippocampus was measured using lucigenin (5 μM bis-N-methylacridinium nitrate, Sigma-Aldrich, St. Louis, MO, USA) enhanced chemiluminescence as previously described [22]. The specific chemiluminescence signal was calculated after subtraction of background activity and expressed as counts per minute per milligram weight (cpm/mg).

Lipid peroxidation of polyunsaturated fatty acid side chains in membrane phospholipids produces the toxic malondialdehyde. We assessed lipid peroxidation levels by measuring the thiobarbituric-acid-reacting substances (TBARs) in homogenates, as previously described [30]. The hippocampal samples were mixed with 1 mL 10% trichloroacetic acid and 1 mL 0.67% thiobarbituric acid. They were then heated in a boiling water bath for 15 min and butanol was added to the solution. After centrifugation, thiobarbituric-acid-reacting substances were determined from the absorbance at 535 nm. The results above were expressed as nmol/g wet tissue.

### Statistical analysis

All data were analyzed using SPSS software (SPSS, Chicago, IL, USA) and one-way analysis of variance (ANOVA). Among-group differences were compared using the least significant *t*-test for homogeneity and Tamhane’s T2 method for heterogeneity of variance. All data were expressed as mean ± SEM, and a *P* value < 0.05 was considered to indicate a statistically significant difference.

## Results

### Characterization of hADSCs

hADSCs used in this study were homogeneous and did not contain endothelial cells or hematopoietic lineages assessed by flow cytometry analysis (Supplementary figure). hADSCs demonstrated acceptable viability (93.5 ± 1.9%).

### Part 1: acute stage (day 3)

No significant differences were found in MI size or hemodynamics among the infarcted groups in the acute stage (data not shown).

#### Biodistribution of intramyocardially administrated hADSCs to the heart and hippocampus

**Fig. 1:**
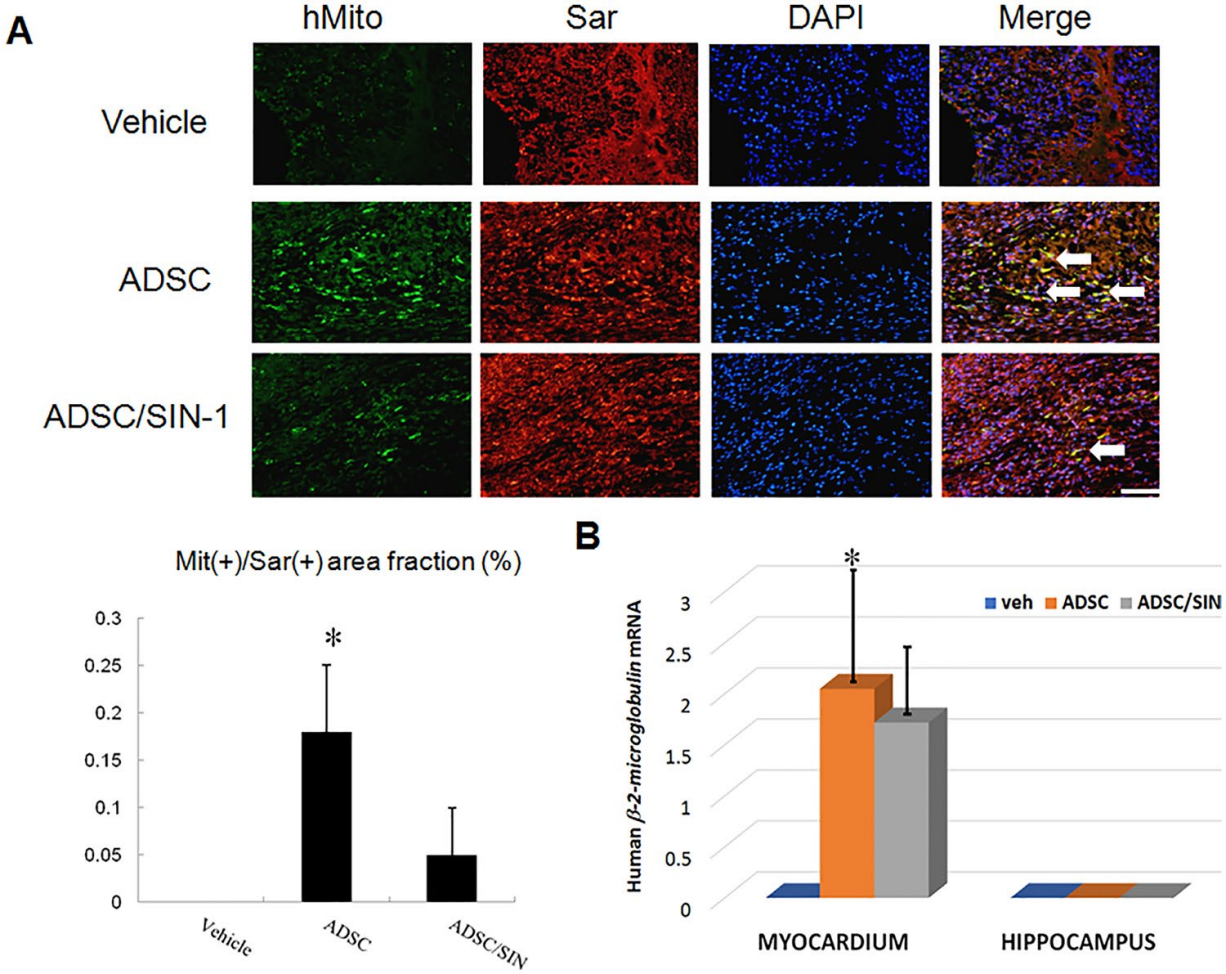
hADSCs identified 3 days after infarction. **A** Immunofluorescent staining results of human mitochondria (green), sarcomeric α-actinin (red), and DAPI (blue). The cardiac phenotype of the transplanted hADSCs was identified from the myocardium border zone. The cardiomyocyte phenotype of transplanted hADSCs was confirmed according to the coexpression of human sarcomeric α-actinin (cardiomyocyte phenotype, red) and mitochondria (green). DAPI was used to visualize nuclei. In the merged image, the coexpression of human sarcomeric α-actinin and mitochondria showed that the antigen expression was colocalized (yellow fluorescence, arrow) (scale bar = 50 μm). **B** qPCR for human *β-2-microglobulin* was performed with sampling from myocardial border zone and hippocampus. No amplification was found in the myocardium treated with vehicle and in the hippocampus of all the groups. *n* = 6 each group. *P < 0.05 compared with infarcted rats treated with vehicle or ADSC/SIN-1

To quantify the total number of transplanted hADSCs in the myocardium, the human origin of these transplanted cells was confirmed by staining with anti-human mitochondria antibodies and the human *β-2-microglobulin* gene 3 days after transplantation. Human stem cells were observed at the implanted areas in the transplantation group but not the vehicle group (Fig. 1A). A few specific clusters of mitochondria-positive human cells were retrieved from the transplanted hearts, and mainly at the border zone. Compared with the infarcted rats treated with hADSCs alone, infarcted rats treated with the combination of hADSCs and SIN-1 showed significantly lower infiltration of the mitochondria-positive cells. qPCR analysis of human *β-2-microglobulin* from the border zone was performed to further confirm that hADSCs were retained in the myocardium. The results showed the presence of human *β-2-microglobulin* sequences in the ADSC recipient myocardium, although only at low levels (Fig. 1B). The fractions of human *β-2-microglobulin* genomic DNA relative to total myocardium DNA were significantly lower in the SIN-1-treated group compared to the ADSCs alone.

As in vivo immunohistochemistry may not be sufficiently sensitive to detect a small number of cells, hippocampi were collected and subjected to qPCR analysis for human *β-2-microglobulin* (Fig. 1B). The results showed no amplification in the hippocampus. Taken together, these results strongly indicated that the remotely transplanted hADSCs had not migrated into the hippocampus from the injection site.

#### Effects of intramyocardial hADSC transplantation on superoxide levels in the circulation, heart, and hippocampus

**Fig. 2:**
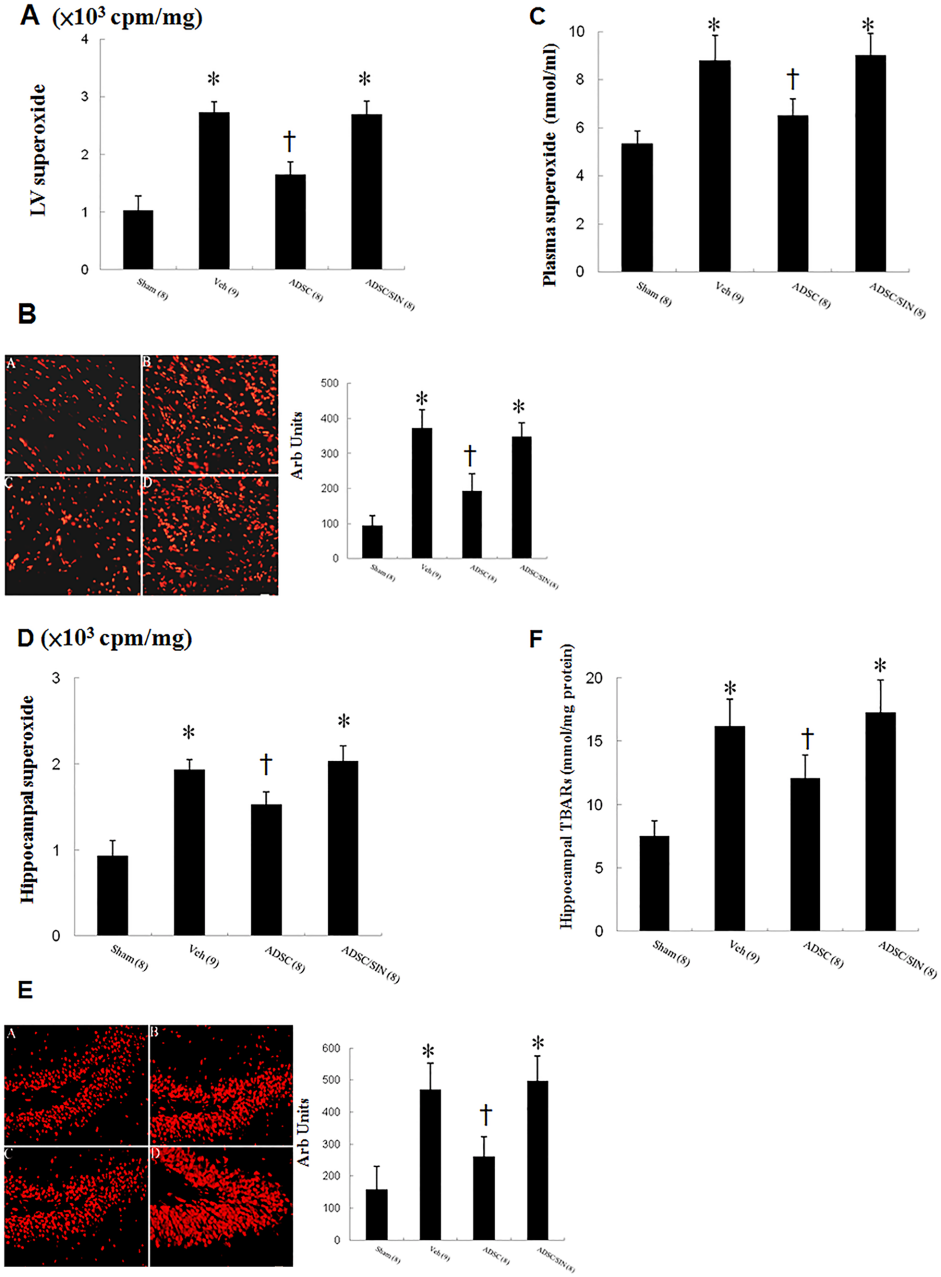
ROS analysis 3 days after infarction. **A** Myocardial superoxide measured by lucigenin-amplified chemiluminescence. **B** DHE staining from the border zone. (A) Sham, (B) vehicle, (C) ADSC, (D) ADSC/SIN-1. **C** Plasma superoxide levels. **D** Hippocampal superoxide measured by lucigenin-amplified chemiluminescence. **E** Hippocampal DHE staining as an index of superoxide stress and quantitative analysis. Hippocampal DHE (red fluorescent) staining showed less intense signals (nuclear position for DHE) in hADSC-treated group compared with vehicle. (A) Sham, (B) vehicle, (C) ADSC, (D) ADSC/SIN-1. **F** Hippocampus lipid peroxidation assessed by TBARs. The number of animals in each group is indicated in parentheses. *P < 0.05 compared with sham and infarcted rats treated with ADSC; †*P* < 0.05 compared with sham

To assess the effects of hADSCs on superoxide anions generated in the myocardium after MI, we performed lucigenin-enhanced chemiluminescence assay. Superoxide production was markedly increased in the myocardium after MI as compared with sham (Fig. 2A). hADSC administration significantly attenuated superoxide levels compared with vehicle. However, SIN-1 reversed the reduced superoxide levels compared with hADSCs alone. The results of lucigenin-enhanced chemiluminescence were further confirmed by DHE staining (Fig. 2B).

Similarly, plasma superoxide levels mirrored the changes of myocardial measurement by lucigenin-enhanced chemiluminescence and DHE reaction (Fig. 2C).

To show the levels of ROS generated in the hippocampus, we performed both methods to detect superoxide anion and TBARs in the tissue (Fig. 2 D, E, F). Compared with sham, vehicle-treated infarcted rats had significantly increased intensity of the fluorescent signal. After hADSC transplantation, the MI rats exhibited a pattern of much lower superoxide levels compared with vehicle.

### Part 2: chronic stage (day 30)

No significant differences in mortality (data not shown) were found among the infarcted groups throughout the study. The relative weights of the hearts at the end of the experimental period corrected for body weight are shown (Table 1). The LV infarcted areas were very thin and had been completely replaced by fully differentiated scar tissue 4 weeks after infarction. There was no significant change in LV weight inclusive of the septum among the infarcted groups during the 4 weeks of the study. However, + dp/d*t* and − dp/d*t* were significantly higher in the hADSC group compared with in the vehicle group, implying the improved cardiac function after administering hADSCs. No significant differences were found in LV end-systolic pressure, LV end-diastolic pressure or infarct size among the infarcted groups.

**Table 1:** Cardiac morphology and hemodynamics 30 days after MI

Parameters	Sham	Infarction		
		Vehicle	ADSC	ADSC/SIN-1
No. of rats	8	9	8	8
Body weight, g	367 ± 12	355 ± 14	372 ± 18	362 ± 16
Heart rate, bpm	392 ± 8	385 ± 12	395 ± 18	389 ± 16
LVESP, mmHg	102 ± 7	97 ± 9	93 ± 7	95 ± 8
LVEDP, mmHg	6 ± 1	18 ± 4*	16 ± 5*	15 ± 4*
+ dP/d*t*, mmHg/s	7141 ± 253	2381 ± 262*	3182 ± 293*†	2283 ± 234*
− dP/d*t*, mmHg/s	6874 ± 288	2152 ± 239*	2872 ± 259*†	2261 ± 192*
Infarct size, %	…	40 ± 3	39 ± 3	39 ± 4
LVW/BW, mg/g	2.48 ± 0.51	3.99 ± 0.62*	3.61 ± 0.58*†	3.77 ± 0.82*

Values are mean ± SD. *BW*, body weight; *LVEDP*, left ventricular end-diastolic pressure; *LVESP*, left ventricular end-systolic pressure; *LVW*, left ventricular weight.

*P < 0.05 compared with sham; †*P* < 0.05 versus vehicle

#### Effect of hADSC transplantation on passive avoidance memory

**Fig. 3:**
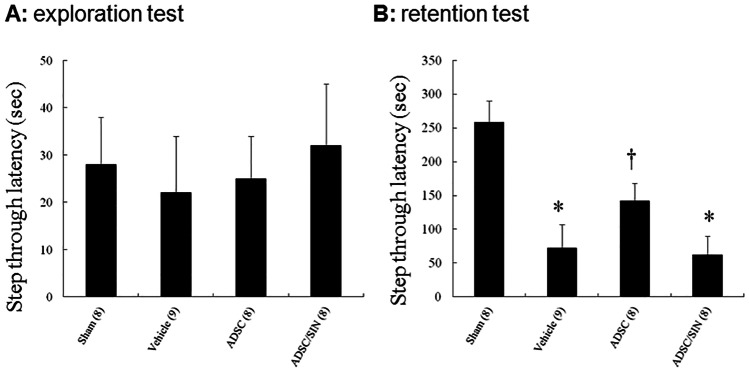
Effects of hADSCs on the step-through latencies in rats 30 days after infarction. **A** Exploration time. **B** Retention time. Each column and bar represent mean ± SEM. The number of animals in each group is indicated in parentheses. *P < 0.05 compared with sham and infarcted rats treated with ADSC; †*P* < 0.05 compared with sham

During the exploration test (on day 1) of the passive avoidance test, infarcted rats showed a similar latency (Fig. 3), implying the homogenous baseline among the groups.

However, in the retention test (on day 2), the step-through latency was significantly shorter in vehicle-treated infarcted rats (72 ± 35 s) than the sham rats (258 ± 32 s) (*P* < 0.05). hADSC administration significantly increased the latency (142 ± 26 s) compared with vehicle (*P* < 0.05). After adding SIN-1, the step-through latency significantly reduced compared with hADSCs alone.

#### Effects of intramyocardial hADSC transplantation on the neurons and spines in the hippocampus

**Fig. 4:**
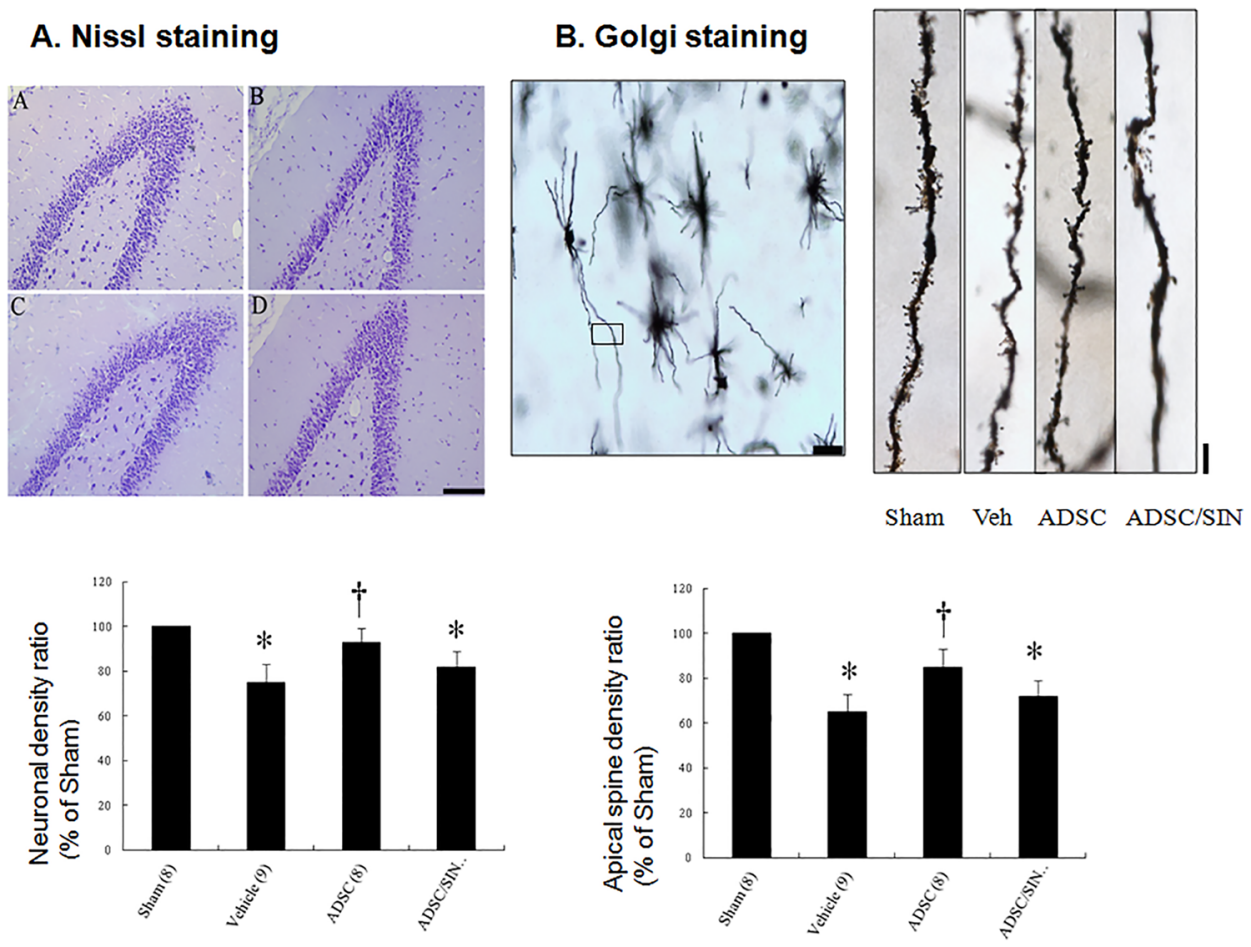
Nissl staining and Golgi stain in rats 30 days after infarction. **A** Nissl staining of the cells in the dentate gyrus region showed an improvement in Nissl granules and darker staining with more ribosomes inside in the ADSC group compared to vehicle. (A) Sham, (B) vehicle, (C) ADSC, (D) ADSC/SIN-1. Scale bar = 100 μm.** B** Golgi stain. Representative neuron morphology with Golgi staining (scale bar = 50 μm) and the framed apical dendritic section for spine density analysis (scale bar = 5 μm). hADSC administration increases the number of dendritic apical spines in dentate gyrus. The number of animals in each group is indicated in parentheses. *P < 0.05 compared with sham and infarcted rats treated with ADSC; †*P* < 0.05 compared with sham

To assess changes in synaptic plasticity, we investigated hippocampal neuronal and dendritic structures using Nissl staining. The total number of surviving neurons in the hippocampal dentate gyrus region was significantly decreased in vehicle group compared with sham (Fig. 4A). Compared with vehicle, rats in the hADSC group had significantly increased neuronal cell survival in the dentate gyrus region of the hippocampus. SIN-1 treatment abolished the beneficial effects on neuronal cell survival compared with hADSCs alone.

We then investigated the formation of dendritic spines in the dentate gyrus using Golgi staining to visualize spine morphology (Fig. 4B). Mean spine density was significantly increased by 30.8 ± 7.4% (*P* < 0.001) in the dentate gyrus of hADSCs compared with vehicle.

#### Effects of intramyocardial hADSC transplantation on synapse number in the hippocampus

**Fig. 5:**
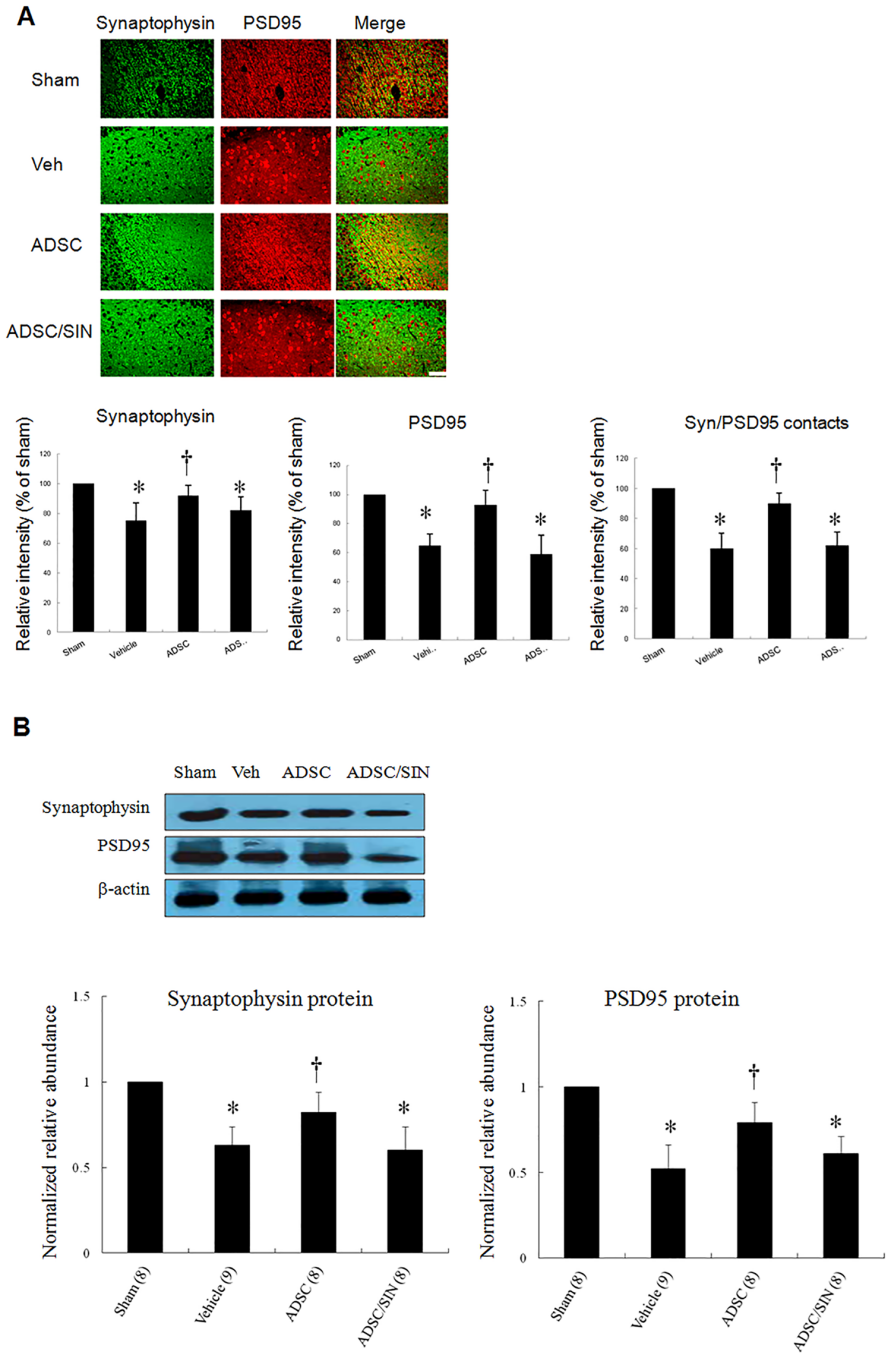
Synaptic plasticity in rats 30 days after infarction. **A** Presynaptic synaptophysin colocalizes with postsynaptic PSD95 in hippocampal dentate gyrus region. Analysis of the relative intensity of synaptophysin, PSD95, and SYN/PSD95 contacts. Quantitative data for synaptophysin and PSD95 show a significantly increased number of synaptophysin and PSD95 stained puncta in the hADSC group compared with vehicle. Importantly, the total number of synaptophysin/PSD contacts (synaptic density) was also increased in the hADSC group. **B** Western blot confirmation for synaptophysin and PSD95 levels in the hippocampus. Quantitative analysis of Western blot analysis. Relative abundance was obtained against that of β-actin. Results are mean ± SEM of 3 independent experiments. The number of animals in each group is indicated in parentheses. Scale bar = 50 μm. *P < 0.05 compared with sham and infarcted rats treated with ADSC; †*P* < 0.05 compared with sham

We next investigated whether hADSCs exhibited alterations in synapse number. The hippocampal dentate gyrus region was subjected to immunohistochemical staining to identify the presynaptic marker synaptophysin and postsynaptic marker PSD95 (Fig. 5A) and visualize pre- and post-synaptic specializations. Intensity of synaptophysin/PSD95 contacts was analyzed as an index of reactive synaptic density, which displayed a 50.1 ± 8.5% increase in the hippocampal dentate gyrus region (*P* < 0.001) of hADSC-treated rats compared with vehicle.

Significant increases in both synaptophysin and PSD95 protein levels were shown by Western blot analysis in the hippocampus of the rats treated with hADSCs (Fig. 5B). Thus, hADSCs resulted in a significant change in protein levels for both markers in the hippocampus. Of note, SIN-1 resulted in impaired synaptophysin and PSD95 protein levels compared with hADSCs alone.

## Discussion

Our results showed for the first time a therapeutic effect of intramyocardial hADSC injection on the brain and heart simultaneously through engagement of the ROS pathway. Mechanistically, the structural recovery and cognitive enhancements elicited by exposure to hADSCs were at least partially mediated by ROS, rather than hADSC engraftment. Taken together, our novel findings suggest that early treatment with hADSCs may decrease the risk of cognitive impairment after MI. The present study was consistent with the notion that functional recovery mediated by transplanted cells could occur without the grafted cells entering the brain if neuroprotective molecules secreted by the cells could reach the injured brain site by crossing the blood–brain barrier.

Our results were consistent with the beneficial effects of intramyocardial administration of hADSCs on cognitive function, as documented structurally by DHE stain, Nissl stain, Golgi stain, and immunofluorescent stain; molecularly by hippocampal synaptophysin and PSD95 proteins; biochemically by plasma superoxide levels; and functionally by passive avoidance test. The beneficial effects of hADSCs on MI-induced cognitive impairment are supported by 4 lines of evidence.

1) ROS in the hippocampus was increased after MI. The present study demonstrates the hippocampal ROS levels were increased after MI, in a correlation with systemic ROS activation. A closer link between the brain and the heart after cardiac damage has been reported than with other nonhematopoietic organs [31]. Damaged cardiomyocytes at the site of MI can increase superoxide anions that rapidly enter the bloodstream. The brain has very low levels of catalase and moderated levels of superoxide dismutase [8]. Thus, even low-grade ROS also substantially affects the blood–brain barrier [32]. Thus, on reaching brain, superoxide regulates acute cytokine response and mediates trafficking of the hippocampus to injured hearts. ROS are increasingly being shown to be intrinsic mediators of communication between the brain and heart.

2) hADSC administration was associated with improved hippocampus-dependent cognitive function after MI. In this study, we used the passive avoidance test to analyze the cognitive and learning ability of the rats to elucidate whether MI could induce cognitive dysfunction. The passive avoidance test has been widely validated as a measure of hippocampus-dependent associative memory function in rodents [33]. A fundamental role in fear memory to plasticity in the hippocampus includes the dentate gyrus [34]. The latency in refraining from entering the compartment in which the rats are electrically shocked has been shown to serve as an index of avoidance ability, through which memory can be assessed. The time required to re-enter the compartment in which the rats are electrically shocked is mostly influenced by learning ability [35]. Thus, the latency time in this study predominantly reflected the memory function of the rats. Our findings indicate that there were memory impairments after MI, but that hADSCs could significantly reverse these memory impairments. Our results support these clinical observations and demonstrate mechanistic links between ROS and post-MI cognitive decline.

3) hADSC administration ameliorated synaptic plasticity via the superoxide radical pathway. The causative effect of ROS on synaptic plasticity has been demonstrated in experimental studies [36]. ROS overproduction suppresses hippocampal neuroplasticity.^35^ Many studies have shown that a reduction of synaptic plasticity was associated with dysfunction in learning and memory performance [37, 38]. A reduction in synaptophysin immunoreactivity has been shown to be accompanied by a decrease in connectivity, which in turn leads to functional deficits in synaptic transmission and eventually cell loss [16, 17]. Since synaptophysin and PSD95 regulated neuronal synaptic activity [13], increases in synapse number assessed by the intensity of synaptophysin puncta colocalized with PSD95 can lead to cognitive improvement. In our experiment, 3 days after MI, we found that the neuron numbers (Nissl stain-positive cells, Fig. 4A), the densities of dendritic spines (Golgi stain-positive cells, Fig. 4B), and the synaptic numbers (co-localization of synaptophysin and PSD95, Fig. 5A) were lower in the vehicle-treated infarcted rats than those in the sham. The impairment can be significantly reversed after adding hADSCs. Furthermore, the addition of SIN-1, a peroxynitrite donor, abolished benefits compared with hADSCs alone, implying that hADSCs may mediate the effects through a superoxide-dependent pathway. Our results were consistent with the findings of Knapp et al. [39], showing that exogenous superoxide administration resulted in transient reduction in postsynaptic response, followed by a late form of long-term potentiation, which can be inhibited by superoxide dismutase application.

4) ROS, not cell engraftment, play a role in mediating neuroprotection. The therapeutic effect of hADSCs is through remote mechanisms. In this study, we used species-specific competitive PCR-based assays to investigate the mechanisms behind the strong behavioral and neurological effects of the hADSCs, as this could yield information regarding how gene families are involved in stem cell migration. Our results showed no amplification in PCR reactions with human *β-2-microglobulin* primer sets at the hippocampus. We proposed the mechanisms through which the remotely distributed hADSCs exert their therapeutic effect by direct antioxidant effect on the hippocampus. As anticipated, reduction in superoxide anion was prevented, and the behavioral and histological protective effects were completely blocked by co-treatment of hADSCs with SIN-1. Our results are consistent with previous studies which showed that stem cells administered intravenously did not reach brain tissue [40], indicating that the benefits of treatment observed for this route of administration are not due to brain stem cell engrafting. 

## Study limitations

First, in the present study, we explored the potential clinical efficacy of hADSCs in the treatment of patients after MI. However, our results using human ADSCs cannot be necessarily extrapolated to rodent ADSCs. There were differential effects between species. Previous studies have shown that the transplantation of human glial progenitor cells into the frontal cortices of immune-deficient neonatal mice has significantly enhanced the cognitive function of adult and aged mice [41]. In contrast, the transplantation of murine glial progenitor cells had no such effects [41]. Such species-specific differences may be explained by the propagation of calcium waves, during which astrocytes can communicate. Human progenitor calcium waves propagate at least three times faster than mouse calcium waves, possibly because they are much larger and have a more complex structure. These differences may also contribute to enhanced long-term potentiation. Second, although it appears that the large accumulation of ROS is necessary for pathological processes to prevail over physiological processes, the levels of tolerance and toxicity have yet to be clarified. Few studies have investigated whether there is a dose-dependent relationship between ROS and plasticity, and future investigations are warranted to investigate this issue. Finally, our study showed that the benefits of intramyocardial injection on cognitive function are not due to brain stem cell engrafting. Our results were consistent with the paracrine hypothesis of stem cells. Stem cells secrete a variety of extracellular vesicles (EVs) that serve as a cell-free therapeutic agent. Mesenchymal stem cell–derived EVs which can cross the blood–brain barrier were enriched in antioxidant miRNAs and exhibited remarkable antioxidant activity evident [42]. Furthermore, EVs prevent cardiac injury by inhibiting inflammation after MI [43]. Comparative analyses of ADSCs and their EVs demonstrated diverse genetic cargo including mRNA and miRNA, and protein contents that play role in angiogenesis, adipogenesis, and regulation of inflammation [44]. Moreover, protein levels and surface markers also differ between EVs and their parent cells [45]. Thus, our results can not be explored into the EVs.

## Conclusions

These data provide new evidence that the intramyocardial administration of hADSCs can systematically regulate the brain and may be a potential intervention for myocardial and cognitive impairment after MI. We identify MI-associated ROS as the cause of cognitive impairment and hADSCs provide mechanistic insights into its reversal. This study may lead to the development of new preventive and therapeutic strategies for MI-related cell therapy.

## Supplementary Information

Below is the link to the electronic supplementary material.

Supplementary file1 (DOCX 1255 KB)

Supplementary file2 (TIF 1520 KB)

## Data Availability

All data are included in the manuscript or in the supplementary material online. All animal procedures were approved by the Animal Care Committee and the hospital ethics committee (approval number: 2018–361).
