# Supplementary material for: Intramyocardial injection of human adipose-derived stem cells ameliorates cognitive deficit by regulating oxidative stress–mediated hippocampal damage after myocardial infarction
Source: J Mol Med (Berl). 2021 Oct 11;99(12):1815–27. doi: 10.1007/s00109-021-02135-6 (PMC8599314; doi:10.1007/s00109-021-02135-6)
Supplement: Supplementary file 1 — Supplementary file1 (DOCX 1255 KB) [file 109_2021_2135_MOESM1_ESM.docx]

**Supplementary Methods**

***Isolation of hADSCs***

hADSCs were purchased from the commercially available kits (Stempro hADSC kit; Invitrogen, Carlsbad, CA, U.S.A) and were generously provided by Gwo Xi Stem Cell Applied Technology (Hsinchu, Taiwan) as previously described (1,2). In brief, hADSCs were cultured in growth media (Low glucose DMEM (Invitrogen) media, 10% FBS (Serana), and 1% Penicillin/Streptomycin (Hyclone)) and were subcultured using TrypLE Express (Invitrogen) to subsequent passages. Passaged cultures will be deemed passage 1. hADSCs at passages 3–5 were used in this study. These hADSCs were homogeneous and did not contain endothelial cells or hematopoietic lineages. Cultured hADSCs have been shown to display mesenchymal stem cell phenotype: they express the mesenchymal stem cell marker CD90 and do not express hematopoietic markers CD31 and CD45. They will be confirmed to be >95% CD90^+^ and <2% CD31^+^/CD45^+^.

***Characterization of hADSC surface phenotype***

Trypsinized hADSCs were suspended in 100 μL PBS. Cells (1×10^5^ per sample) were treated at room temperature for 20 min with the following specific anti-human antibodies: anti-Isotype IgG1-PE, -CD19-PE, -CD34-PE, -CD73-PE, -CD105-PE, -Isotype IgG1-FITC, -CD45-FITC, -CD90-FITC, -Isotype IgG2a-PE, -CD14-PE, -HLA-DR-PE (BD Biosciences, San Jose, CA, USA). Mouse IgG was used as a negative control condition. Fluorescent labeling was analyzed with a flow cytometer (Accuri C6; BD Biosciences, San Jose, CA, USA; supplementary figure). The cell quality has been approved to be clinical-grade by the authority in Taiwan as we previously described (1). Besides, the hADSCs prepared by Gwo Xi Stem Cell Applied Technology have been used in clinical trial for liver cirrhosis and stroke (Topic: Clinical Trial Study About Human Adipose-Derived Stem Cells in the Liver Cirrhosis, ClinicalTrials.gov Identifier**:** NCT02297867; Clinical Trial Study About Human Adipose-Derived Stem Cells in the Stroke, ClinicalTrials.gov Identifier**:** NCT02813512).

***Hemodynamics and infarct size measurements***

Hemodynamic parameters were measured in anesthetized rats at the end of passive avoidance test (30^th^ day after MI). A polyethylene Millar catheter was inserted into the LV and connected to a transducer (Model SPR-407; Miller Instruments, Houston, TX, USA) to measure LV systolic and diastolic pressure as the mean of measurements of five consecutive pressure cycles as previously described (2). The maximal rates of LV pressure rise (+dP/d*t*) and decrease (−dP/d*t*) were measured. After the arterial pressure measurement, the atria and the right ventricle were trimmed off, and the LV was rinsed in cold physiological saline, weighed, and immediately frozen in liquid nitrogen after a coronal section of the LV obtained for infarct size estimation. Each section was stained with hematoxylin and eosin and trichrome. The infarct size was determined as previously described (2). With respect to clinical importance, only rats with large infarction (>30%) were selected for analysis.

***In situ detection of superoxide anion in the heart and brain***

Intracellular superoxide production in the dentate gyrus and myocardium regions was evaluated using *in situ* dihydroethidium (DHE, 1 µM, Invitrogen-Molecular Probes, Eugene, OR, USA). Fluorescent paraffin-embedded tissue sections (5 µm) were incubated with DHE in PBS (10 mM) at room temperature in a humidified, dark container for 30 minutes. Superoxide radicals generated by the tissue subsequently formed ethidium, a red fluorescent compound, in nuclei. The density of fluorescence was expressed as arbitrary units/mm^2^ field.

***qPCR of human-specific β-2-microglobulin***

To trace whether hADSCs migrated into the hippocampus, quantitative PCR was used by specific primers for human sequence for β-2-microglobulin with the TaqMan system (Prism 7700 Sequence Detection System, PE Biosystems) as previously described (2). The samples were obtained from the hippocampus at the 3th day after MI. The oligonucleotide primers of β-2-microglobulin are human-specific as the housekeeping gene absent in the rat, which has been confirmed previously (3). We used hADSC-treated rats from the myocardial border zone (< 2 mm within the infarct) as positive controls, which have been shown to retain hADSCs by immunohistochemistry. Vehicle served as negative control. For quantification, *mRNA* expression was normalized to the expressed housekeeping gene *β-actin* with gene sequences shared by human and rats.

For *human β-2-microglobulin,* the primers were 5′-CCAGCAGAGAATGGAAAGTC-3′ (sense) and 5′-GATGCTGCTTACATGTCTCG-3′ (antisense).

For *β-actin,* the primers were 5′-TTCTACAATGAGCTGCGTGTG-3′ (sense) and 5′-GGGGTGTTGAAGGTCTCAAA-3′ (antisense).

A computer linked to a detector was used to control the reaction conditions for 40 cycles of the amplification step.

Supplementary figure


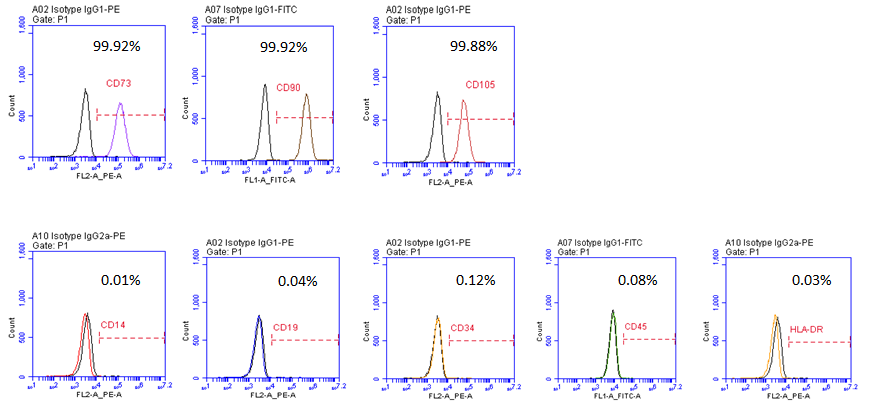


**References**

1. Lee TM, Harn HJ, Chiou TW, Chuang MH, Chen CH, Chuang CH, Lin PC, Lin SZ. [Remote transplantation of human adipose-derived stem cells induces regression of cardiac hypertrophy by regulating the macrophage polarization in spontaneously hypertensive rats.](https://pubmed.ncbi.nlm.nih.gov/31164286/) Redox Biol. 2019;27:101170.

2. Lee TM, Harn HJ, Chiou TW, Chuang MH, Chen CH, Lin PC, Lin SZ. [Targeting the pathway of GSK-3β/nerve growth factor to attenuate post-infarction arrhythmias by preconditioned adipose-derived stem cells.](https://www.ncbi.nlm.nih.gov/pubmed/28130118) J Mol Cell Cardiol. 2017;104:17-30.

3. [van der Pluijm](https://pubmed.ncbi.nlm.nih.gov/?term=van+der+Pluijm+G&cauthor_id=11393785) G, [Sijmons](https://pubmed.ncbi.nlm.nih.gov/?term=Sijmons+B&cauthor_id=11393785) B, [Vloedgraven](https://pubmed.ncbi.nlm.nih.gov/?term=Vloedgraven+H&cauthor_id=11393785) H, [Deckers](https://pubmed.ncbi.nlm.nih.gov/?term=Deckers+M&cauthor_id=11393785) M, [Papapoulos](https://pubmed.ncbi.nlm.nih.gov/?term=Papapoulos+S&cauthor_id=11393785) S, [Löwik](https://pubmed.ncbi.nlm.nih.gov/?term=L%C3%B6wik+C&cauthor_id=11393785) C. Monitoring metastatic behavior of human tumor cells in mice with species-specific polymerase chain reaction: elevated expression of angiogenesis and bone resorption stimulators by breast cancer in bone metastases. J Bone Miner Res 2001;16:1077-1091.
