# Supplementary figures and images for: Intramyocardial injection of human adipose-derived stem cells ameliorates cognitive deficit by regulating oxidative stress–mediated hippocampal damage after myocardial infarction
Source: J Mol Med (Berl). 2021 Oct 11;99(12):1815–27. doi: 10.1007/s00109-021-02135-6 (PMC8599314; doi:10.1007/s00109-021-02135-6)

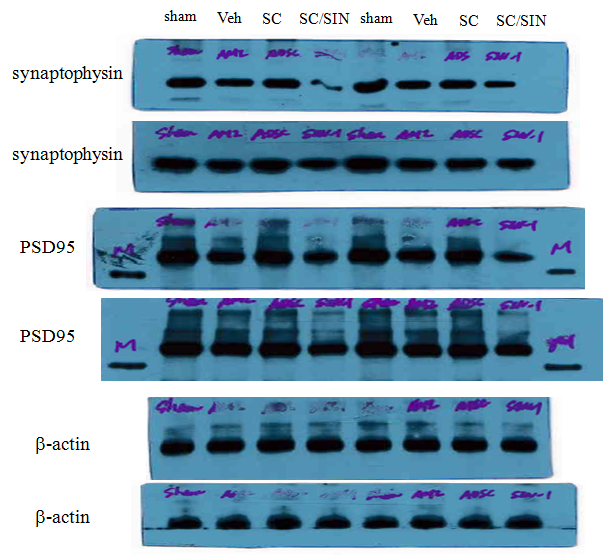

Supplement: Supplementary file 2 — Supplementary file2 (TIF 1520 KB) [file 109_2021_2135_MOESM2_ESM.tif]
